# BergaCare SmartLipids: commercial lipophilic active concentrates for improved performance of dermal products

**DOI:** 10.3762/bjnano.10.208

**Published:** 2019-11-04

**Authors:** Florence Olechowski, Rainer H Müller, Sung Min Pyo

**Affiliations:** 1 Berg + Schmidt GmbH & Co. KG, An der Alster 81, 20099 Hamburg, Germany; 2 Freie Universität Berlin, Institute of Pharmacy, Pharmaceutics, Pharmaceutical Nanotechnology & NutriCosmetics, Kelchstr. 31, Berlin 12169, Germany

**Keywords:** chemical stabilization of active agents, controlled release, firm inclusion, nanostructured lipid carriers (NLCs), penetration enhancement, skin occlusion, SmartLipids, solid lipid nanoparticles (SLNs)

## Abstract

SmartLipids are the latest generation of dermal lipid nanoparticles with solid particle matrix. Their characteristic properties resulting from the “chaotic” and disordered particle matrix structure are reviewed. These properties are high loading and firm inclusion of active agents, physical stability of the particle matrix lipid modification (primarily α, β′), and related to these three properties the improved chemical stabilization of labile active agents. Exemplarily data for these effects are shown and underlying mechanisms are discussed. Further, general properties of lipid nanoparticles, which are also exhibited by the SmartLipids, are reviewed. These include the restauration of the protective lipid skin barrier (anti-pollution effect), penetration enhancement by occlusion (invisible patch effect) and the option to control the release of active agents for optimized biological effect and reduction of side effects (e.g., skin irritation through sensitizing active agents), which improves the skin tolerability. Regulatory aspects, such as submicron particle status, excipients, and certifications, are also discussed.

## Introduction

To meet the increasing expectations and demands of consumers in personal care and cosmetics, as well of patients in medical care, dermal delivery systems are needed to improve the performance of dermal formulations. There are also requirements from the formulation manufacturer regarding perfected delivery systems. The primary requirements for an optimized carrier system are:

1. Sufficiently high loading capacity allowing for the utilization of low amounts of carrier, so as to not distinctly affect the properties of the formulation, e.g., skin feeling;
2. physical stability, i.e., firm inclusion of active agents and remaining integrity of the carriers in the final formulation during shelf life;
3. effective protection and stabilization of chemically labile active agents, e.g., retinol as a classical example;
4. restoration of the natural skin barrier (recently in focus in the framework of “anti-pollution” strategies);
5. penetration enhancement and increased bioavailability allowing for a reduced application frequency, enabling the use of active agents that could not be used before due to lack of penetration, e.g., certain antioxidants;
6. controlled and prolonged release in order to avoid too high concentrations on the skin that may cause irritancy (e.g., retinol and tretinoin);
7. composition preferentially of natural or naturally derived and modified raw materials that are ideally biodegradable and in conformity to ECOCERT/COSMOS (e.g., natural lipids or semisynthetic lipids derived from natural lipids).

Emulsions can only partially meet these requirements. For example, the protection of chemically lipophilic active agents is limited by the diffusional exchange with water. Controlled release is not possible due to the high diffusion coefficient, *D*, in oils of low viscosity (Einstein equation). Release typically takes place very fast within seconds or milliseconds [1]. The age of smart delivery systems for skin started with the introduction of liposomes to the cosmetic market in 1986 from the company Dior with the product Capture. Liposomes have several advantages for dermal delivery, e.g., adhesiveness to skin due to the small nanometric size, increase of skin moisture related leading to wrinkle reduction, and a stabilization of active agents to some extent.

In the following years attempts were made to come up with better delivery systems of the next generation. Looking back, these efforts were of limited or no success. For example, many expectations were raised with dermal microemulsions. However, the need of relatively high surfactant concentrations (skin irritating/damaging effects) and the often unpleasant application feeling did lead to a market failure. Polymeric nanoparticles, developed by P. P. Speiser for pharmaceutical purposes in the middle of the 1970s [2], found only limited use in consumer care/cosmetics. Problems are often the lack of regulatory status of many polymers used, expensive large-scale production and the lack of biodegradability (no “green” products possible, thus no certification after ECOCERT/COSMOS). There were also developments of various “somes”, being derived from the liposomes and finding few applications (e.g., niosomes, ethosomes, transfersomes, pharmacosomes, herbosomes, colloidosomes, sphinosomes and cubosomes [3].

A step forward in 1991 was the development of a carrier made from solid lipids, the solid lipid nanoparticles (SLNs). They are derived from the emulsions by replacing the liquid lipid (oil) with a solid lipid and therefore are solid at body temperature. The second generation of these particles, the nanostructured lipid carriers (NLCs), made it to the cosmetic market in 2005 and are typically a mixture of one solid lipid and one liquid lipid [4], e.g., tristearin and caprylic/capric triglyceride, also being solid at body temperature.

In 2014 a new generation of carriers was developed, called SmartLipids [5–6]. They combine all the existing advantages of the previous particles made from solid lipids/lipid blends and add new key features. These are distinctly increased loading capacity and firm inclusion of the active agents, together with improved physical stability and increased chemical stabilization. This was achieved by creating a “chaotic” matrix structure by blending many different lipids, e.g., up to ten solid lipids, or mixture of solid and liquid lipids [7]. The structure of the SmartLipids and related key advantages are discussed. Briefly reviewed are the properties of SmartLipids originating from being solid lipid particles, i.e., possessing the same beneficial properties as abovementioned SLNs and NLCs. Industrial aspects such as regulatory issues and technical questions are covered regarding the production of marketable products in cosmetics and consumer care.

## Review

### What exactly are SmartLipids – definition

SLN were made from typically one solid lipid only. Such lipids can form highly ordered lipid crystalline structures (β modification), which leaves little space to accommodate active agents and limits its loading capacity. Sometimes expulsion of the active agent takes place during storage, when the lipid re-orders from α and/or β′ modification to primarily β modification. To overcome the loading limitation in NLCs, oil was admixed to the solid lipid, since liquid lipids (oils) exhibit higher solubilities for active agents compared to solid lipids. In NLCs, the solubilities of solid and liquid lipid are approximately additive, i.e., the solubility of active agents in the NLC particle blend is increased. However, the increase comes at the expense of an accelerated reordering during storage, as oils can accelerate the transition process to the β modification.

**Figure 1:**
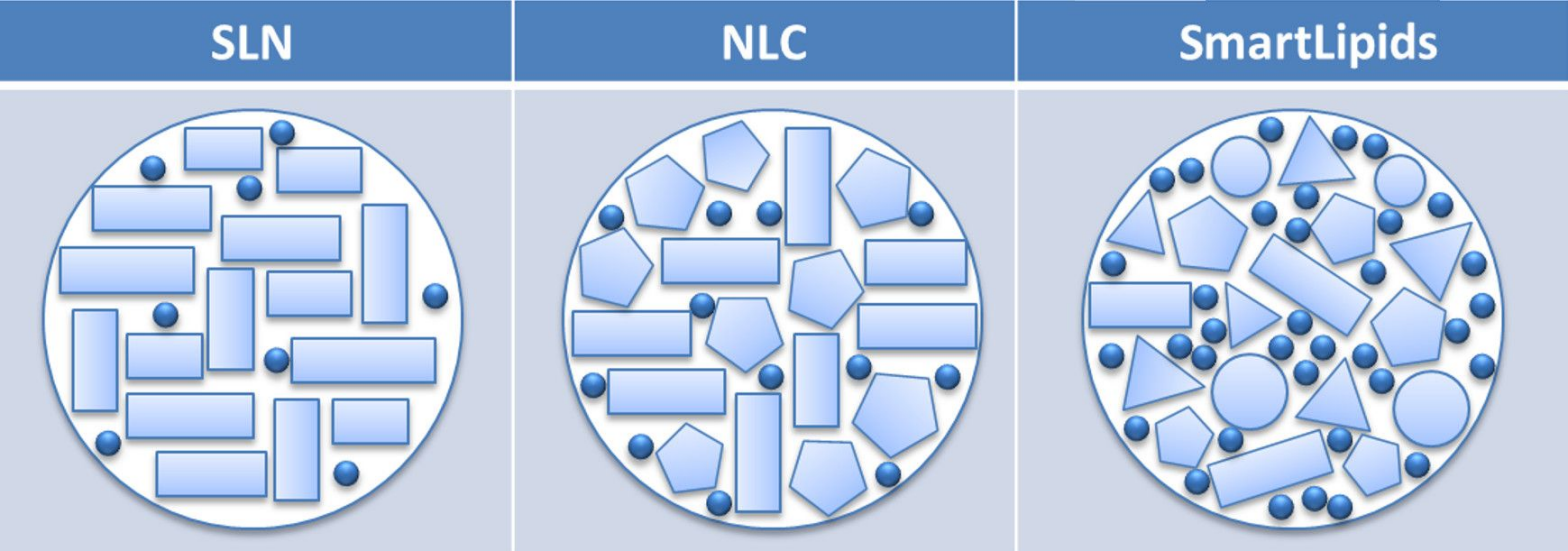
Simple representation of the structure of SmartLipids. The mixture of structurally very different solid and liquid lipids leads to a chaotic, less ordered structure (α, β′) with many imperfections, providing high loading capacity. The structure remains unchanged during storage leading to a firm inclusion of active agents with chemical stabilization and no expulsion of the active agents [10].

In the SmartLipids principle many structurally very different lipids (typically up to ten) are blended on purpose, being spatially incompatible due to differences in structure (mixture of mono-, di and triglycerides, or waxes) and length of the fatty acid chains. This was achieved by blending solid lipids only (e.g., with chains C14–C22), or by admixing limited amounts of oils (e.g., containing C8–C12 fatty acids). This leads to a lipid particle matrix structure being primarily or to a considerable extent in α modification with some β′ modification, having many imperfections for an enhanced loading capacity of active agents (Figure 1). Due to the structural differences of the lipid molecules, reordering during storage to the highly ordered β modification is blocked or distinctly slowed down. Thus, no expulsion of active agents takes place leading to firm inclusion. The addition of oils increases the loading capacity further, but oil addition needs to be limited in order to prevent an accelerated reordering of the lipid matrix.

Some of the previously described NLC particles already represent the “SmartLipids principle”. This is the case when the one solid lipid used in the mixture of one solid/one liquid lipid is a commercial lipid that is already a blend of multiple lipids, or when it is a priori a “wild mixture” of different structures, e.g., carnauba wax, mostly consisting of aliphatic esters (40.0 wt %), diesters of 4-hydroxy cinnamic acid (21.0 wt %), ω-hydroxy carboxylic acids (13.0 wt %) and fatty acid alcohols (12.0 wt %). The chain length of fatty acids and alcohols is mainly in the range of C26–C30 [8]. Another classical example of a commercial lipid blend is Cutina LM, which is composed of three pure lipids and three natural lipid mixtures (cetearyl alcohol, cetearyl glucoside, octyldodecanol, carnauba wax, candelilla wax, and beeswax). Different from the SmartLipids principle are, e.g., NLCs made from a relatively uniform solid lipid, e.g., tristearin (mainly C18 triglyceride) blended with caprylic/capric triglyceride [9].

### Dermal application – main key features of SmartLipids

#### High loading capacity and firm inclusion of active agents

**Figure 2:**
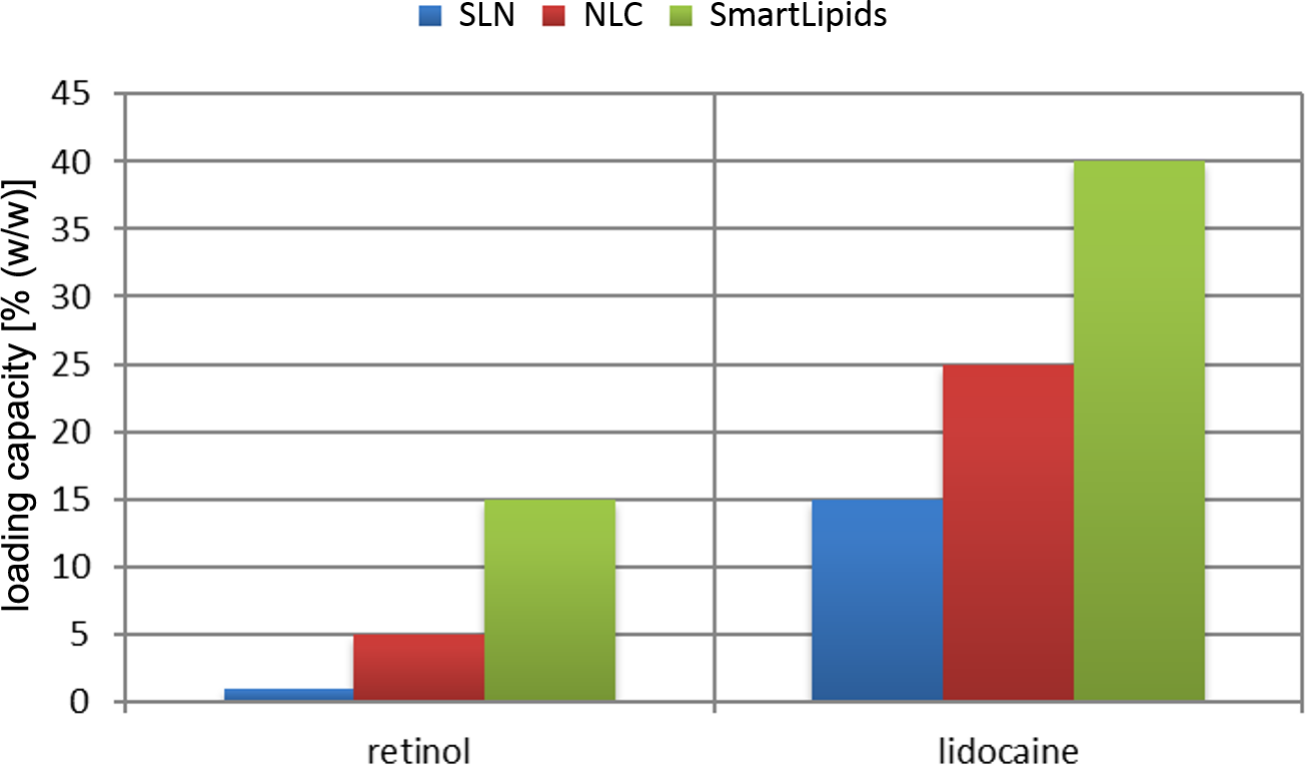
Maximum loading reported for SLNs, NLCs and SmartLipids for the dermal active agents retinol [11–13] (left) and lidocaine [14] (right).

The unordered “chaotic” matrix state of SmartLipids provides enough defects to accommodate distinctly higher amounts of active agents compared to SLNs and NLCs. This can be seen regarding the maximum loading achieved for retinol and lidocaine (Figure 2). In this case the loading increases by a factor of about 15 and 3, respectively.

The higher loading capacity ensures the firm inclusion of the active agent as well. In NLCs it was sometimes necessary to incorporate, e.g., 5% retinol (= maximum loading) to achieve the desired retinol concentration in the final product. At the maximum loading capacity, reordering could cause the expulsion of the active agent into the water phase (crystal formation). This is not the case in SmartLipids, since the maximum loading capacity is increased to 15. Even slight reordering will not lead to active expulsion when lower loadings than the maximum possible are employed. Of course, the degree of loading affects also the release (cf. section 3.4).

#### Physical stability in products

**Figure 3:**
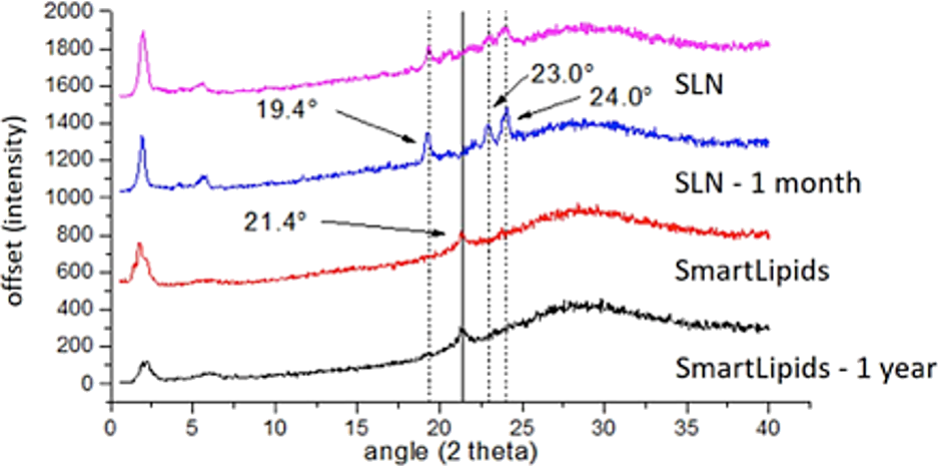
X-ray diffraction patterns of SLNs (pink curve) and SmartLipids mixture (red curve) determined directly after production and after one month (SLNs: blue curve) and one year (SmartLipids: black curve) storage at room temperature [5].

The aspects of physical stability in lipid particle dispersions during storage and in final products are (1) an unchanged crystalline structure of the particle matrix and (2) the quantitative proof of remaining existence/presence of particles. The created crystalline structure should primarily remain in the unordered state for firm inclusion of loaded active agents, which can be measured by combining differential scanning calorimetry (DSC) with X-ray diffraction. Ruick showed a fast transition from the α modification to the β modification when SLNs were produced with tristearin (Figure 3), while a SmartLipids mixture with eight solid lipids remained practically unchanged during one year of storage.

Carriers, especially fluid carriers such as liposomes, can reduce in number in a final product during shelf life. Liposomes have a tendency to fuse with the stabilizer layers of oil droplets in oil/water creams. For the best quality, the quantitative stability of the carriers is desirable. Especially, quantitative stability is required in case of pharmaceutical formulations. Quantitative analysis of, e.g., liposomes in final products is relatively difficult, but required in some countries from regulatory authorities. The difficulty of such analysis was tried to circumvent, e.g., by only specifying “lecithin” in the product, despite knowing that a main contribution of the product performance is coming from the existing liposomes formed by lecithin. In contrast, the quantitative proof of 100% existing lipid particles with solid particle matrix is very easy by measuring the melting enthalpy using differential scanning calorimetry (DSC). The melting enthalpy of a SmartLipids suspension can be determined before addition to the product, after addition to a gel or a cream and after storage. In case the particles dissolve, e.g., in an oil of a cream, the melting enthalpy will decrease. Thus, the physical stability of SmartLipids is easy to analyze and to prove.

#### Chemical stabilization of active agents

**Figure 4:**
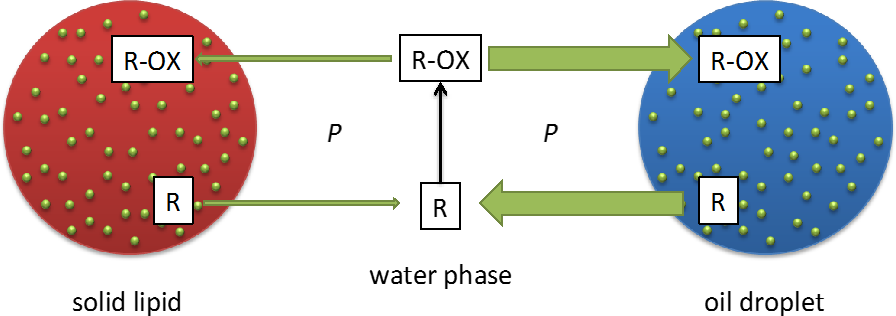
Fast degradation of lipophilic active agents (R) in fluid carriers (blue), such as oil/water emulsions or creams and liposomes, through fast exchange with the water phase (right) in comparison to a minimized exchange of active between a highly viscous solid carrier (red) and the water phase (left) (*P*: partitioning coefficient); modified with permission from [17], copyright 2017 Elsevier.

Classical fluid carriers such as nanoemulsion and liposomes have a limited ability to protect labile lipophilic active agents. Due to the partitioning coefficient, *K*, after Nernst, the lipophilic active agents are primarily enriched in the lipid phase and only to a small extent in the water phase. But there is a diffusional exchange between the oil and the water phase. The active agent diffuses from the oil into the water phase, the active agent is degraded in the water phase (e.g., hydrolyzed), the degraded active agent diffuses back into the oil phase and is replaced in the water phase by new non-degraded active agent from the oil. This is a kind of vicious circle (Figure 4, right). Due to the relatively low viscosity, η, of oil (70–100 mPa·s) and phospholipid bilayers (184 mPa·s [15]), this process takes place relatively fast. The diffusion coefficient *D* can be calculated using the Einstein equation:


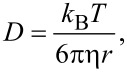


with *k*_B_ being the Boltzmann constant and *T* being the absolute temperature. In contrast, the viscosity η in solid particles is very high, e.g., 653 mPa·s for solid paraffin. Thus, the abovementioned exchange is practically avoided or at least strongly minimized [16] (Figure 4).

**Figure 5:**
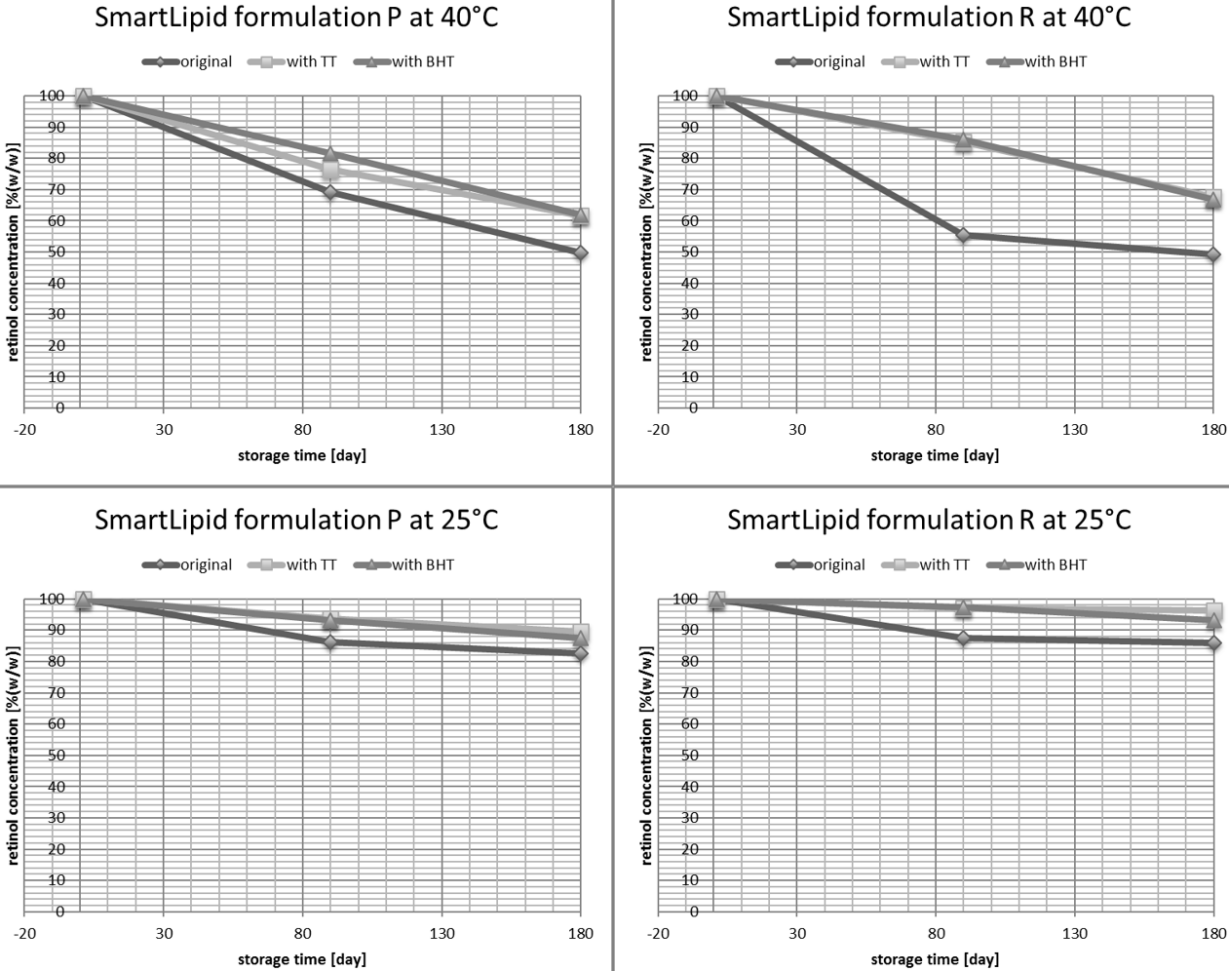
Storage study with retinol loaded SmartLipids particles over six months. Remaining retinol content in SmartLipids formulation P (left) and R (right) before and after incorporation of lipophilic antioxidants Tinogard TT and BHT at 40°C (upper) and room temperature (25°C, lower), [10].

Retinol is a chemically highly labile molecule, and thus the efficient stabilization in dermal products is a challenge. Retinol was incorporated into various carrier systems, but only with limited increase in stability. Stabilities reported were 20% after ten days in liposomes [18], 40% after 24 h in a nanoemulsion [19], 50% after 24 h in zein colloidal particles [19] and 60% after one month in a nanoemulsion stabilized by silica [20]. A screening was performed in two SmartLipids mixtures. The solid particle matrix itself is protective for labile molecules as outlined above. Up to 86% retinol remained after six months of storage at room temperature (Figure 5, lower right). This stabilization is due to the solid character of the carrier matrix with limited exchange of active agent with the water phase, and the protection of the active agent inside the solid carrier matrix, e.g., against access of oxygen and light. To have an even better stabilized system, lipophilic anti-oxidants were added leading to about 85–95% retinol remaining at room temperature (Figure 5, lower row), and 65–70% at a stress temperature of 40 °C (Figure 5, upper row). More complex particle structures of SmartLipids lead to better incorporation and protection of active agents.

#### Size of SmartLipids submicrometer particles – legal aspects

By definition, nanoparticles possess a size in the nanometer range, i.e., from a few nanometers to below 1000 nm. Legally, after the EU cosmetic guideline [21], nanoparticles are only particles with a size below 100 nm, precisely a particle population with more than 50% of the particles below 100 nm based on the number distribution. Incorporating a nanoparticle material needs to be declared in the INCI nomenclature by the addition of “nano”.

A typical mean size of SmartLipids particles is in the range of 200–400 nm, measured by laser light scattering, i.e., photon correlation spectroscopy (PCS). The calculated size is based on the intensity signal of the scattered laser light, which is used to calculate a so-called correlation function *g*(τ). The obtained mean diameter is the intensity-weighted so-called *z*-average (*z*-ave). The correlation function *g*(τ) can be converted to a size distribution by Fourier transformation. EU regulations require a measuring method that can cover the full size range of a particle population. The measuring range of PCS is roughly from about 3 nm to 6 µm. Suspensions of SmartLipids do not contain particles below 3 nm, and practically no, or only negligible contents of, particles larger than 6 µm. Thus, PCS is a suitable method to meet the EU requirements for SmartLipids products. The absence of particles above 6 µm could be proven by laser diffraction (Malvern Mastersizer 2000, Malvern, UK). In this case the volume distribution was taken, because it is more sensitive to show a few large particles than the number distribution.

To avoid the linguistic confusions: technically the particles are nanoparticles but at the same time are legally not nanoparticles. Those particle populations should be classified as “submicrometer particles”. Submicrometer particles are in the size range between 100 nm and 1000 nm, nanoparticles are smaller than 100 nm. Hence, SmartLipids are submicrometer particles.

#### Industrial production of SmartLipids - standard & customized products

The production of SmartLipids is identical to that of SLNs and NLCs [22]. The lipid mixture is heated to approximately 5–10 °C above the melting point of the highest melting lipid, then the active agent is dissolved in the lipid melt and the melt containing the active agent is dispersed in a hot aqueous stabilizer solution (surfactant, polymer) of identical temperature by high-speed stirring to form a coarse emulsion. This pre-emulsion is then passed through a high-pressure homogenizer, and typically one or two homogenization cycles are applied. Homogenizers used are typically piston–gap homogenizers, e.g., the APV Gaulin 5.5. homogenizer with a production capacity of up to 150 L/h (medium scale) [7], and the GEA Niro Soavi homogenizer with production capacities of up to 10.000 L/h (large scale). The obtained hot nanoemulsion is cooled, the lipid blend re-crystallizes and forms solid lipid particles. The suspensions are preserved by standard preservatives (e.g., euxyl^®^ PE 9010) or are alternatively prepared preservative-free by adding, e.g., pentylene glycol. Standard products are available on the market, while coenzyme Q10 and retinol are in preparation. Formulations with customer-specific active agents can also be prepared on an exclusive basis. Concentrates have a typical particle content of 10% or 20%. For the incorporation into cosmetic products, the dermal formulations are produced as normal, but with slightly reduced water content. At the end of the production, the SmartLipids concentrates are admixed under blending with a stirrer.

### Dermal application – general features of lipid nanoparticles with solid particle matrix

#### Adhesion onto skin with increased residence time/prolonged release

There are many properties that are identical for the several types of lipid nanoparticles – SLNs, NLCs and SmartLipids – because they only depend on physical characteristics (e.g., particle size or general adhesiveness of small particles) or chemical characteristics (e.g., nature of the particle material, in this case lipid). Thus, data published previously regarding these general features also apply to the newly developed SmartLipids particles.

**Figure 6:**
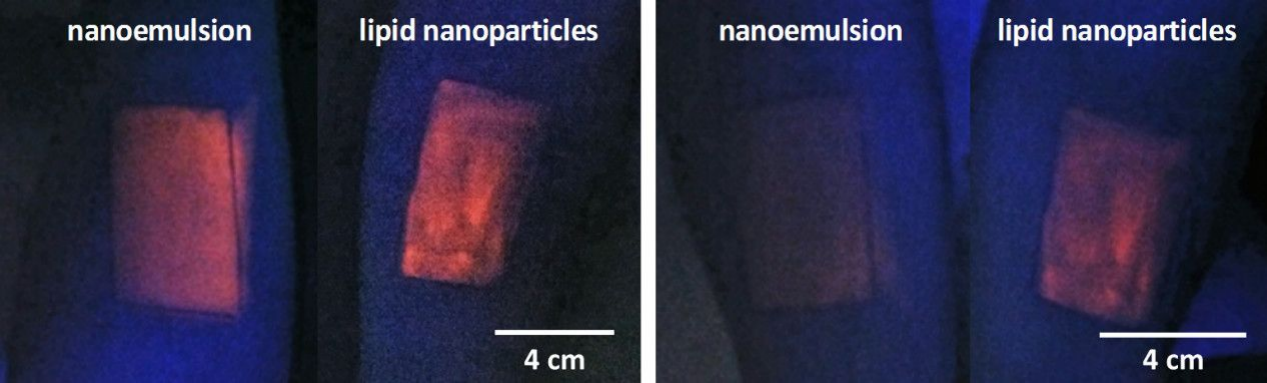
Left: Nile red-labeled lipid nanoparticle suspension (right arm) and nanoemulsion (left arm) applied to human forearm directly after application. Right: **B**oth arms after washing under flowing water with rubbing. The lipid nanoparticles are highly adhesive and mostly remain on the skin (right arm).

It is well known that decreasing the particle size leads to a larger interaction area between particles and substrate and thereby the adhesiveness increases. The classical and often cited example from food industry is the different adhesiveness of crystalline sugar and icing sugar onto bakery products. Large crystals tend to fall off, whereas icing sugar forms sticky layers on bakery products. Thus, all nanoparticles, e.g., as reported for liposomes [23–24], have an increased adhesiveness to skin. In a recent study it could be shown that the adhesiveness of solid lipid particle suspensions to human skin is superior to fluid nanoemulsion droplets. Both, similar in size, were labeled with Nile red and applied in a square onto the inner forearm. The presence of applied particles was detected using an UV lamp with a wavelength of 365 nm (Figure 6, left). Then the forearms were washed under flowing water and rubbed with the thumb. Most of the nanoemulsion was washed off, whereas the lipid particles mostly remained on the skin (Figure 6, right). This increased adhesiveness and related residence time on the skin promotes a prolonged release of active agents, especially when sweating occurs with subsequent tissue wiping by the consumer or when skin areas are covered with clothes.

#### Restauration of the natural skin barrier and anti-pollution effect

Due to unhealthy skin conditions or general stress by environmental factors, the natural skin barrier (lipid film on the stratum corneum) can be damaged, i.e., thinned or even partially lost (bare patches). This has negative effects on the cells below and could lead to a higher transepidermal water loss (TEWL). In addition, the skin is more exposed to environmental stress, ranging from UV/IR radiation to pollution from the air, e.g., particulate matter. Especially in the last two years, skin care products/cosmetics with “anti-pollution” effect are a key topic. Cosmetic formulations can reduce the adsorption of particulate matter onto the skin and minimize related negative skin effects. Thus, the effective restauration of the skin barrier is an important feature of lipid nanoparticles [25], and a long residence time of the particles on the skin even under mechanical stress is beneficial to fight pollution factors.

**Figure 7:**
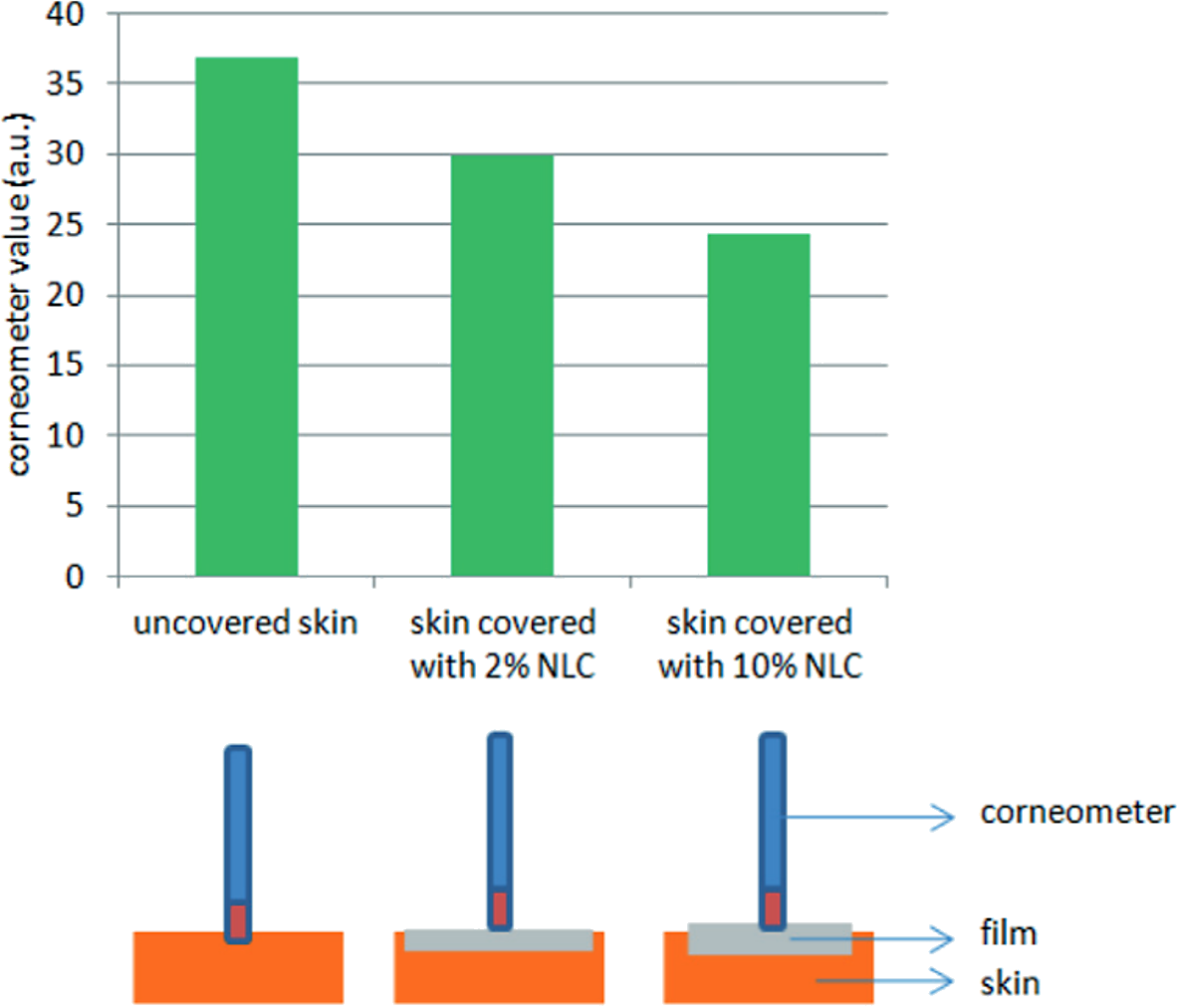
Determination of the relative film thickness by measuring the dielectric constant *D* on skin. Probe readings of untreated skin and skin with applied increasing concentration of lipid particle suspension; reproduced with permission from [27], copyright 2013 Euro Cosmetics.

The lipid particles adhere onto the skin as any nanosized particle does and form a film. This film formation can be followed by measuring the dielectric constant of the skin, using a Corneometer^®^ (Courage + Khazaka Electronic GmbH, Germany) [26]. The probe determines the dielectric constant, *D*, of the skin through a condenser in the probe. An insulator medium in the condenser reduces the measured *D* value. For example, *D* is 0 for a complete insulator (vacuum), about 5 for lipids and organic liquids, and 80 for pure water. The reading is given in arbitrary units. If the skin moisture increases through the application of a cosmetic cream, the obtained *D* value increases. However, if an insulator material is between the probe and the skin (i.e., a lipid film), the *D* value decreases (Figure 7, lower row). This was shown in [27–28]. Applying a lipid particle film to the skin led to the formation of a lipid layer thicker than the normal barrier, resulting in *D* values below the *D* values of the normal lipid film on skin (Figure 7, upper row). The thickness of the created lipid film by applying lipid particle suspensions is controlled by the particle concentration in the suspension. The formation of a film can also be concluded by measuring the decrease in transepidermal water loss (TEWL) after application of the lipid particles in a formulation.

Following this model, any lipid particle can be used to repair the stratum corneum film, independent on its lipid composition and loaded active agent, as it was the case in [27–28]. Restauration of the natural lipid barrier is thus a beneficial effect generated already by unloaded SmartLipids on the skin. Also, ceramides can be used to produce SmartLipids, albeit primarily for the higher priced cosmetic sector.

#### Increased penetration and bioavailability

Occlusion increases the dermal penetration of many active agents. Occlusion leads to increased moisture content of the skin as well as increased percutaneous absorption of most active agents [29–30]. A simple but efficient way to obtain occlusion in the hospital is to cover a cream-treated skin area with a plastic foil. Pharmaceutical transdermal patches benefit also from the occlusion effect of their polymer films, promoting penetration. However, such patches are of no or limited use for facial cosmetics. A compromise is to apply cosmetic masks for a limited time, requiring consumer patience and compliance to the regular treatment. In contrast, lipid particle formulations are described in the literature as “invisible patch” [27]. After being applied to the skin, they show controlled occlusive and delivery effects comparable to patches (or some masks) but are “invisible” and thus create their effect up to 24 h. Increased penetration of active agents due to occlusion is extensively described in the literature [29].

**Figure 8:**
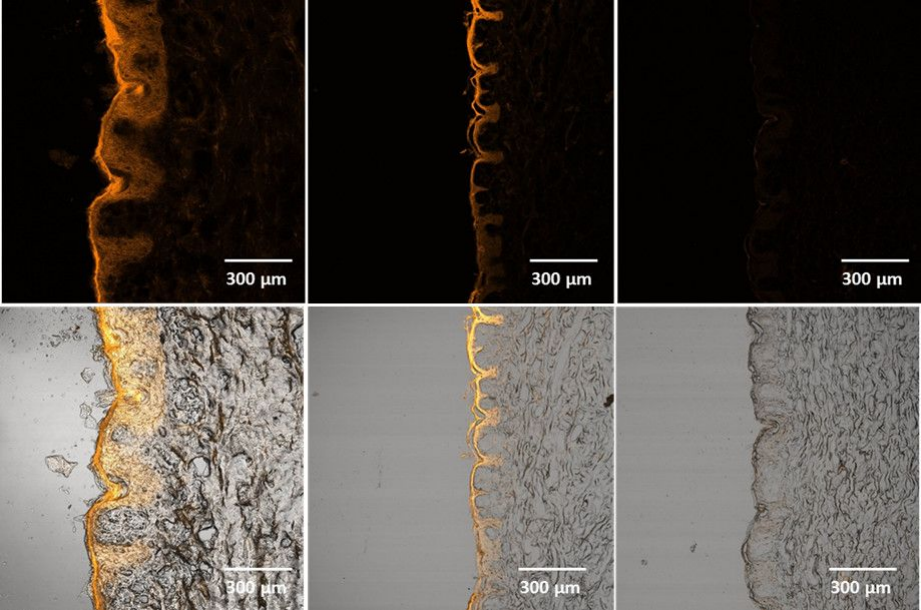
Penetration of curcumin into pig ear skin, vertical pig ear slices, fluorescence microscopy images (upper row) and overlay of fluorescence and light microscopy images (lower row); after application of 0.2% curcumin suspension in SmartLipids (left column), 2.0% curcumin micrometer-size crystal suspension (middle) and a commercial curcumin containing gel product (right). Incubation time was 24 h. Both laser strength (35%) and fluorescence detector gain (720 V) were kept constant during all measurements.

To show the ability of SmartLipids to provide efficient skin penetration, a particle suspension was prepared containing 0.2% curcumin. Curcumin has many positive effects on the skin [31–32] and is at the same time fluorescent, allowing for a good and easy detection in the skin by fluorescence microscopy. The suspension was applied to pig ear skin in a covered Franz cell, incubated for 24 h and then skin slices were investigated by normal light and fluorescence microscopy (Figure 8, left column). Figure 8 (upper row) shows the fluorescence microscopy images. The lower row shows overlays of fluorescence and light microscopy images, allowing to locate the fluorescence in the epidermis. For comparison, a tenfold higher concentrated 2% curcumin suspension is shown in Figure 8 (middle column). The curcumin remains as a thick fluorescent layer on top of the stratum corneum, showing practically no fluorescence inside the epidermis. Finally, a curcumin containing marketed product from the US is shown, according to HPLC analysis containing 0.0001% curcumin in dissolved form, corresponding to its maximum solubility. Only negligible fluorescence in the skin was detectable (Figure 8, right column). This shows that SmartLipids are an enabling technology to provide efficient skin penetration even for such problematic, poorly soluble active agents such as curcumin. This increased bioavailability in the skin enables real skin effects.

This study based on curcumin fluorescence was a qualitative study as the fluourescence intensity was not measured quantitatively. However, strong fluorescence (SmartLipids), limited fluorescence (suspension) and practically no fluorescence (marketed product) show clear differences. The comparison with the marketed product was meant to show that there is an urgent need for improved dermal curcumin formulations. It should be kept in mind, that both the SmartLipids and the micrometer-crystal formulation contained a mixture of non-dissolved curcumin and curcumin dissolved in the outer phase of the dermal formulation. The non-dissolved curcumin acts as a depot to replace the curcumin in the formulation penetrating the skin. This is the way, how the formulations work, and the SmartLipids work better.

#### Controlled and prolonged release

Optimized performance of active agents might depend strongly on the rate of their release from the carrier. Desirable release might be fast, prolonged/sustained or should be as slow as possible (even to no release at all). That means, a carrier system should provide the ability to control the release of active agents. A prolonged release is desirable in many formulations, e.g., in anti-aging compounds such as retinol. A controlled release is also desirable for active agents having a skin irritation potential, because concentrations that are too high and promote irritation need to be avoided. It was shown for tretinoin, that incorporation into lipid particles avoided skin irritation [33]. Little or no release is ideal for molecular sunscreens for UV protection, which should not penetrate into but remain on the skin, ideally inside the carrier, to generate their protective effect. At best, they should remain inside the lipid particle, since it was shown that there is a synergistic effect of the particle matrix on the molecular sunscreen increasing its protective efficiency [34]. This allows the reduction of molecular sunscreen concentration in a product.

**Figure 9:**
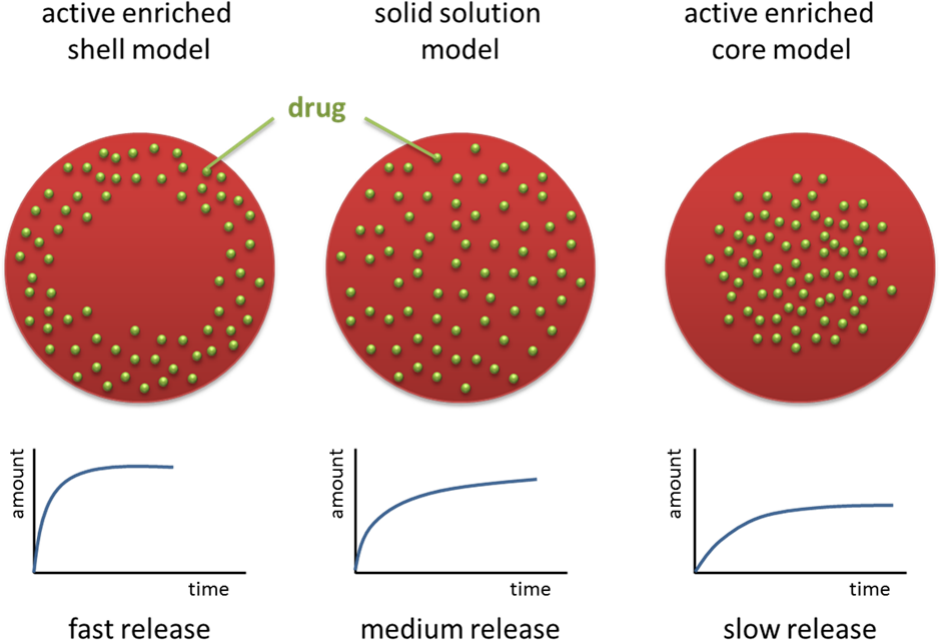
Models of cosmetic active agents or drugs (green) incorporated into lipid nanoparticles, from left to right: enriched shell model (fast release), solid solution model (prolonged release) and enriched core model (very slow release); reproduced with permission from [17], copyright 2017 Elsevier.

**Figure 10:**
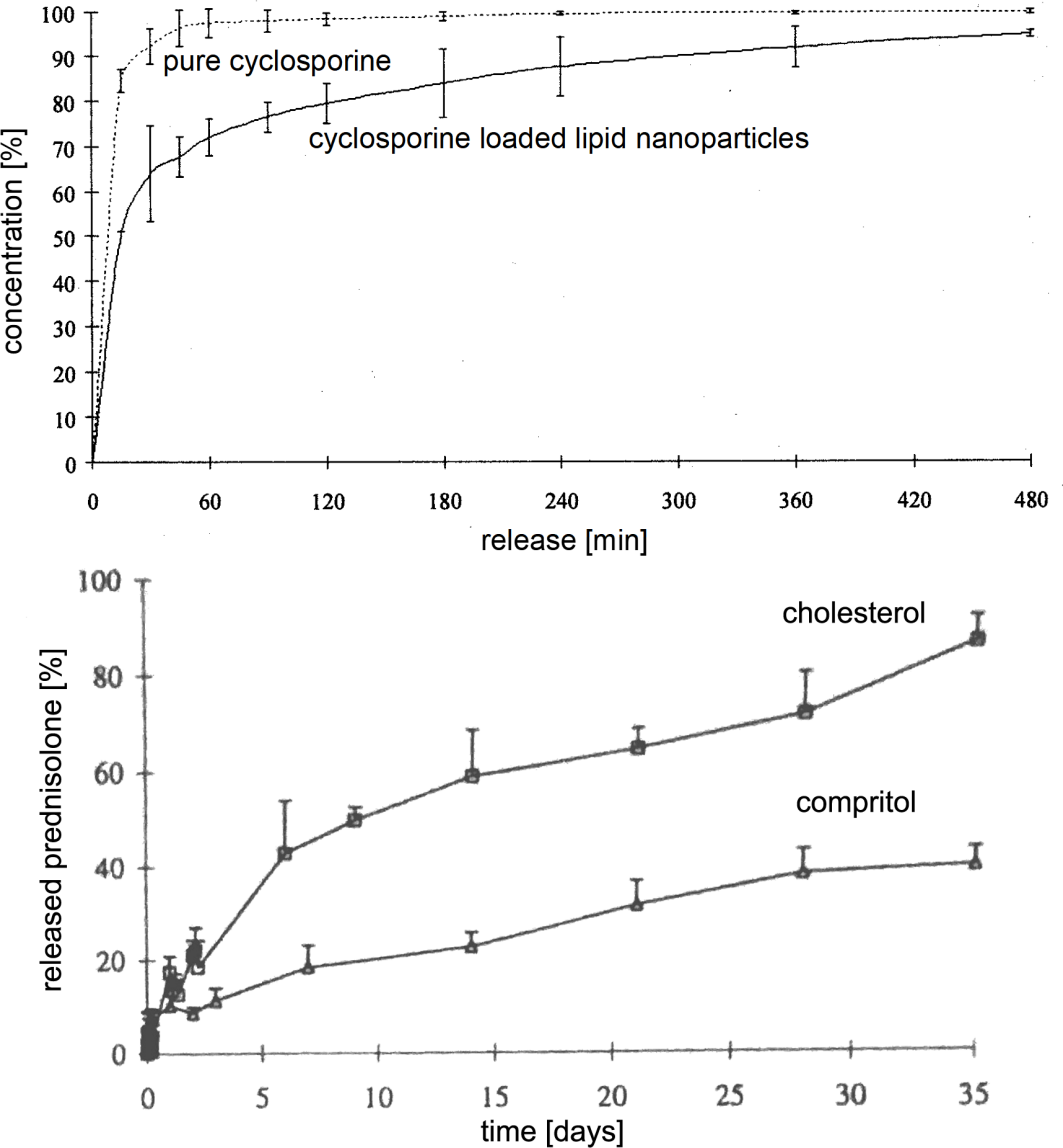
Examples of release profiles from lipid nanoparticles: very fast release of cyclosporine within minutes (upper row, after [35]), and the other extreme of very slow release of prednisolone over 35 days (lower row, after [36], explainable by enriched shell and enriched core model, respectively.

The release can be controlled by the localization of the active agent inside the solid lipid particle matrix. Without going into technical details, the localization of the active agent can be modified by the composition of the lipid particle matrix, concentration of active agent and production parameters. A very fast release is generated, when the active agent is primarily located in the outer shell of the particles (enriched shell model), a prolonged release when the active is evenly distributed throughout the whole carrier matrix (solid solution model), and a very slow/strongly delayed release when the active is mainly located in the core (enriched core model) (Figure 9). For example, a very fast and complete release within minutes was shown for cyclosporine (Figure 10, upper row) [35], and an extremely prolonged release of only 37.1% of prednisolone after 5 weeks was achieved with solid lipid nanoparticles (Figure 10, lower row) [36] The distinctly reduced side effects of tretinoin released from lipid nanoparticles can be explained by a controlled release, avoiding high peak concentrations of free tretinoin on the skin.

**Figure 11:**
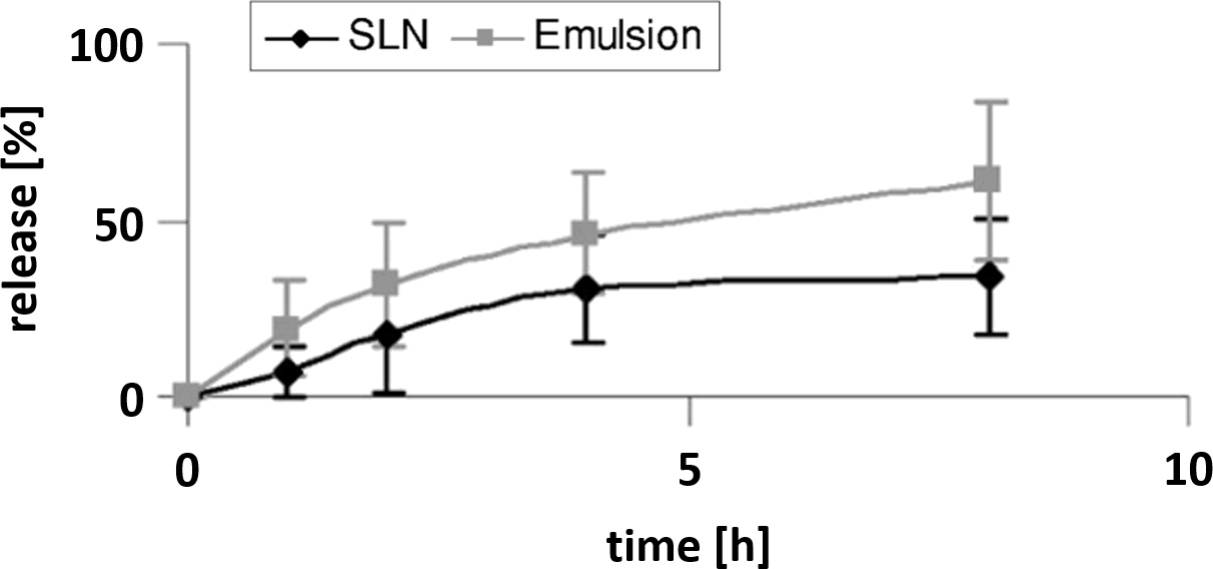
In vitro release of the sunscreen oxybenzone from a nanoemulsion (grey) and from a SLN suspension (black) measured in Franz diffusion cells, after [37].

In dermal sunscreen formulations, the lipophilic sunscreens are normally dissolved in the oil phase of oil/water emulsions. Due to the liquid state of the oil droplets, the evenly and molecularly dispersed sunscreen within the droplets can be released quickly and penetrate into the skin. The release of oxybenzone from an emulsion and SLNs was compared in an in vitro Franz cell model. The release from emulsions was two times faster (Figure 11) [37]. The distinctly slower release from the lipid nanoparticles shows their potential of reducing undesired side effects by large amounts of sunscreen penetrating into the skin.

## Conclusion

The lipid nanoparticle history started in 1991 with SLNs, the second generation of NLCs entered the cosmetic market just 14 years later in 2005, much faster than the liposomes did (they needed about 20 years from invention to cosmetic market in 1986). With the “SmartLipids concept” a mature industrial delivery system is available since 2016. The outstanding feature is the number of delivery advantages combined in one single system. Specific to SmartLipids are:

1. High loading capacity,
2. firm inclusion of active agents,
3. physical stability of the carrier in the final formulation (and easy to prove) and thus
4. improved chemical stabilization of active agents.

General features of all lipid nanoparticles are the restauration of the natural protective lipid barrier, and penetration enhancement with increased dermal bioavailability and controlled/optimized release.

Even if a different carrier system is superior in one or two properties, the sum of the combined delivery advantages in SmartLipids outweighs this in most cases. On top, to meet upcoming consumer expectations, conformity to ECOCERT/COSMOS can be provided and the particles are not nanosized but submicrometer carriers. Contract manufacturing on industrial scale under GMP is available, also for customized formulations. Thus, the basis for broad use in cosmetics and consumer care products is given.
